# The Psychobiological Problems of Continued Nicotine Dependency in E-Cigarette ‘Vapers’. Commentary: “Electronic Cigarettes”

**DOI:** 10.3389/fpsyt.2015.00123

**Published:** 2015-09-01

**Authors:** Andrew C. Parrott

**Affiliations:** ^1^Department of Psychology, Swansea University, Swansea, UK

**Keywords:** nicotine, E-cigarette, addiction, health, psychobiology

Electronic cigarettes were originally introduced as devices to facilitate smoking cessation, but their efficacy in this regard has not been established. Instead, they are often used to facilitate continued cigarette smoking in adults, and are common entry devices for nicotine dependency in children and adolescents (1). The aim of this commentary is to emphasize these problems since far from improving health, E-cigarettes may paradoxically be damaging physical/psychological health. Furthermore, while this is certainly an issue for active smokers, it may become an increasing problem for those who are “passively vaping” E-cigarettes.

E-cigarettes are battery-operated devices, which deliver hits of nicotine via the inhalation of an aerosol spray, which contains a mixture of nicotine, flavorings, and other chemicals (1–3). The E-cigarette was originally introduced as an aid to facilitate smoking cessation. It followed the successful development of earlier nicotine substitution devices, such as nicotine chewing gum, nicotine transdermal patch, and the inhaler (4–6). However, these earlier products were only partially effective at nicotine delivery, with 2 mg gum producing around 25% of the psychophysiological effects of a nicotine cigarette and 4 mg nicotine gum producing around 40% of its physiological effects (7). Yet, despite their comparative inefficacy at nicotine delivery, they partially assuaged nicotine cravings, which allowed them to double the success rates for smoking cessation (4). Crucially, this was achieved *without* making these early nicotine substitution devices attractive to non-smokers.

E-cigarettes were consciously designed to make them attractive, but unfortunately this can now be seen as a mistake since it has encouraged their use as a facilitator for continued smoking in adults, and has introduced many youngsters into nicotine and nicotine dependency. Grana et al. (1) note that they were designed to look like cigarettes; also they were given “kid-friendly flavors, including grape, chocolate, bubble gum, and gummy bear”; furthermore, they have also been marketed “aggressively” often using simplistic and misleading messages. Since first introduced into the USA and Europe in 2006, their usage has risen dramatically, with sales doubling every year [Wells Fargo Internet report cited in Ref. (3)] so that instead of being used to aid tobacco cessation, they are often being used to facilitate continued cigarette smoking. Grana et al. (1) noted that many adult smokers continue to smoke tobacco in private, but vape E-cigarettes where smoking is banned. Furthermore, E-cigarettes are providing a simple introduction into nicotine dependency for children and adolescents. Grana et al. (1) reported that youth use of E-cigarettes had increased from 3.3% in 2011 to 6.8% in 2012. They further noted that around a third of adolescent E-cigarette users have never smoked a tobacco cigarette. Hence, another major concern is the potential progression to higher self-dosing with nicotine – readily achieved by progressing onto cigarette smoking.

Grana et al. (1) noted several of health problems associated with E-cigarettes: the noxious cancerous chemicals in the aerosol vapors, the dangers of passive vaping (viz: as with passive cigarette smoking), and several other problems. However, they did not describe one of the most important problems – that nicotine is highly addictive drug, and that regular nicotine usage has many damaging psychological consequences. In neuropsychobiological terms, nicotine is a powerful CNS stimulant, with many basic similarities to other CNS stimulants, such as cocaine and methamphetamine. Hence, the acute effects of increased heart rate, greater alertness, and mood intensity are similar to every other CNS stimulant drug. All drugs in this class generate brief mood gains, but they are soon followed by negative moods (feeling tired and stressed), during the post-drug recovery period [for review, see Ref. (8)]. With nicotine, this mood fluctuation can be very rapid, with moods fluctuating up and down in parallel with their nicotine intake [viz: every 20–30 min in regular smokers; see Figure 1 in Ref. (9)]. This rapid mood fluctuation helps explain its high addiction potential. Indeed, nicotine is one of the most addictive of all drugs due to its very rapid onset and consequent downturn. Hence, nicotine is similar to cocaine in its psychobiological effects and addiction profile (10). Furthermore, as with every CNS stimulant drug, nicotine can damage the integrity of the HPA axis. Hence, all stimulant drugs can lead to deficits in homeostasis, with disrupted sleep, disrupted circadian rhythms, altered cortisol levels, and other neurohormonal deficits [for review, see Ref. (8)]. Regular nicotine users can also suffer from neurocognitive and other psychobiological deficits, which I have outlined in earlier reviews (11, 12). These included daily mood fluctuation, increased stress, heightened depression, poorer memory, and neuroimaging data indicative of other neurocognitive deficits (6, 13–15). Finally, these psychobiological problems tend to be greater in disadvantaged individuals with a propensity for distress, making nicotine and other stimulant drugs particularly damaging vulnerable individuals (12).

In summary, nicotine is a powerful CNS stimulant with a wide range of adverse effects. Its addictive potential may be widely recognized, but there is far less realization about its damaging psychobiological and health effects – on heart rate, mood stability, alertness, neurocognitive skills, sleep, the HPA axis, cortisol, and stress (12). These psychobiological deficits are likely to be found in regular users of E-cigarettes, even in those who use them alone (i.e., without smoking tobacco cigarettes in parallel; see Table 1). The main aim of this commentary is to emphasize that all these core functions, which are crucial for human well-being, need to be empirically studied in E-cigarette users. My core prediction is that these damaging effects of nicotine in cigarette smokers will be replicated in users of E-cigarettes.

**Table 1:** **The adverse effects of nicotine dependency in cigarette smokers, and the similar psychobiological problems predicted for E-cigarette users**.

*Mood fluctuation*: smokers typically experience positive moods on smoking, followed by negative moods on nicotine withdrawal. This mood vacillation is rapid, with moods going up and down every day in parallel with cigarette/nicotine intake (9, 11). Similar types of mood fluctuation are likely to be found in regular E-cigarette “vapers”
*Psychophysiological vacillation*: the repetitive mood changes of smokers are just one element of a core psychobiological fluctuation. Hence, alertness goes up and down over the day, in parallel with the changes in mood state. Cognitive skills and memory abilities may also fluctuate (13). Similar core psychobiological fluctuations may also develop with E-cigarette users
*Addiction potential*: psychobiological fluctuation provides an essential rationale for nicotine’s strong addiction potential. Similar basic processes underlie the addiction potential of cocaine (10) and other CNS stimulants, such as methamphetamine, MDMA, and mephedrone (8). To the extent that E-cigarettes are effective at nicotine delivery – they will also show addiction potential
*Wider psychobiological problems*: nicotine dependency in cigarette smokers leads to greater daily stress, depression, cognitive deficits, worse memory, poorer sleep, lower self-efficacy, and many other deficits (12–15). Similar psychobiological problems are predicted to develop in regular uses of E-cigarettes

## Conflict of Interest Statement

The author declares that the research was conducted in the absence of any commercial or financial relationships that could be construed as a potential conflict of interest.
